# A Dual-Polarized and Broadband Multiple-Antenna System for 5G Cellular Communications

**DOI:** 10.3390/s25041032

**Published:** 2025-02-09

**Authors:** Haleh Jahanbakhsh Basherlou, Naser Ojaroudi Parchin, Chan Hwang See

**Affiliations:** School of Computing, Engineering and the Built Environment, Edinburgh Napier University, Edinburgh EH10 5DT, UK; c.see@napier.ac.uk

**Keywords:** 5G, broadband communications, CPW-fed slot antennas, dual-polarized antenna, mmWave phased arrays, smartphone applications

## Abstract

This study presents a new multiple-input multiple-output (MIMO) antenna array system designed for sub-6 GHz fifth generation (5G) cellular applications. The design features eight compact trapezoid slot elements with L-shaped CPW (Coplanar Waveguide) feedlines, providing broad bandwidth and radiation/polarization diversity. The antenna elements are compact in size and function within the frequency spectrum spanning from 3.2 to 6 GHz. They have been strategically positioned at the peripheral corners of the smartphone mainboard, resulting in a compact overall footprint of 75 mm × 150 mm FR4. Within this design framework, there are four pairs of antennas, each aligned to offer both horizontal and vertical polarization options. In addition, despite the absence of decoupling structures, the adjacent elements in the array exhibit high isolation. The array demonstrates a good bandwidth of 2800 MHz, essential for 5G applications requiring high data rates and reliable connectivity, high radiation efficiency, and dual-polarized/full-coverage radiation. Furthermore, it achieves low ECC (Envelope Correlation Coefficient) and TARC (Total Active Reflection Coefficient) values, measuring better than 0.005 and −20 dB, respectively. With its compact and planar configuration, quite broad bandwidth, acceptable SAR (Specific Absorption Rate) and excellent radiation characteristics, this suggested MIMO antenna array design shows good promise for integration into 5G hand-portable devices. Furthermore, a compact phased-array millimeter-wave (mmWave) antenna with broad bandwidth is introduced as a proof of concept for higher frequency antenna integration. This design underscores the potential to support future 5G and 6G applications, enabling advanced connectivity in smartphones.

## 1. Introduction

The continuous evolution of wireless systems has driven demand for higher data rates and improved services. As a result, 5G wireless communication has emerged to tackle the challenges posed by the limitations of the current generation’s (4G) cellular networks [1,2]. It is poised that 5G technology can transform wireless connectivity by delivering a wide array of services, including enabling the IoT, enhancing mobile broadband, and offering ultra-reliable communication systems. Its ability to integrate these capabilities positions 5G a critical enabler for the next generation of connected devices and services [3]. One of the key technologies driving the advancements in 5G networks is MIMO systems. MIMO technology utilizes multiple antennas at both the transmitting and receiving ends, enhancing communication reliability and significantly increasing channel capacity. By leveraging multiple signal paths, MIMO improves data throughput and efficiency in wireless communication systems, making it a key component in modern wireless networks. While MIMO technology has been extensively utilized in 4G LTE systems, it plays an even more pivotal role in 5G and 6G networks. By increasing the number of radiation elements, MIMO systems in 5G can achieve even higher performance levels. This makes it a highly efficient and promising solution for meeting the increasing demands of data-heavy applications [4]. In addition to its reliability and capacity benefits, MIMO technology also employs diversity schemes in antenna configurations. This involves sending the same signals with uncorrelated antennas, which helps combat fading and significantly improves the reliability of wireless links. By leveraging the benefits of MIMO technology and diversity schemes, 5G networks are poised to deliver seamless connectivity and cater to a wide array of emerging applications.

Multiple antennas in the system enable advanced techniques like MIMO, beamforming, and spatial diversity, paving the way for efficient data transmission, improved link reliability, and overall performance enhancements in 5G smartphones [5,6]. The focus on broadband capabilities is another key attribute enabling seamless connectivity across a wide frequency range [7]. In addition, by incorporating dual polarization, this antenna system allows the simultaneous transmission and reception of signals with both horizontal and vertical polarizations, significantly enhancing data throughput and mitigating potential interference issues. This capability is especially crucial in dense urban environments and areas with high network congestion, ensuring a stable and reliable connection for smartphone users [8]. As a result, 5G smartphones equipped with this technology can efficiently adapt to diverse network deployments, regulatory environments, and varying user requirements, ensuring a consistent and high-quality user experience. Moreover, printed antennas are becoming increasingly popular in cellular communications. Their compact size and simple design contribute to efficiency and ease of integration into mobile devices. Among various printed antennas, omnidirectional antennas such as monopole, slot, loop, and PIFA are predominantly employed in MIMO smartphone systems, as they offer superior omnidirectional coverage and wider bandwidth. These characteristics ensure comprehensive performance across different sides of the mainboard. In contrast, directional antennas like patch antennas are less commonly used due to their larger size and limited radiation coverage [9].

In recent years, various smartphone MIMO antenna designs have been reported to cater to the needs of sub-6 GHz cellular networks [10,11,12,13,14,15,16,17,18,19,20,21,22,23,24,25,26,27,28,29,30,31,32,33,34,35]. However, many existing smartphone antennas face challenges, such as narrow operational bandwidths, single-polarized radiators, or uniplanar designs that occupy substantial PCB space and increase system complexity. To address these challenges, we introduce a new 5G MIMO antenna featuring four-element/eight-port radiators with a CPW feeding technique, ensuring a quite broad impedance bandwidth and facilitating polarization/pattern diversity functionality. CPW-fed antennas and microwave components have gained significant popularity in wireless applications due to their appealing characteristics, including compact size, conformal shape, lightweight composition, ease of fabrication, and seamless integration. In comparison to probe/microstrip-fed antennas, CPW-fed antennas offer the advantage of achieving wideband impedance matching effortlessly [36,37]. Moreover, the suggested design comprises four pairs of compact antennas positioned at the four edges of the smartphone’s board featuring dual-polarization characteristics. Realized on an inexpensive FR-4 substrate, each element incorporates a trapezoid slot antenna complemented by an L-shaped microstrip stub. To enhance the isolation between adjacent slot antennas, the arrow-shaped strip between antennas serves as a decoupling structure. The compact antenna elements efficiently operate within the frequency range of 3.2–6 GHz, exhibiting dual-polarization functionality and providing full radiation coverage, ensuring comprehensive connectivity. The novelty of the proposed MIMO smartphone antenna lies in its development as a compact, wideband, and dual-polarized antenna array designed to overcome key limitations of existing designs. Unlike most of the reported antennas with limited bandwidth, this array offers an extended frequency range of over 2800 MHz, effectively covering a broad spectrum of sub-6 GHz frequencies. In addition, its planar and compact design allows for seamless integration into a single-layer PCB, reducing system complexity. Furthermore, the array features dual-polarized radiation, enhancing coverage and performance across various device orientations. These innovations provide superior functionality, distinguishing the proposed antenna from other sub-6 GHz MIMO smartphone antenna designs.

In addition to the sub-6 GHz spectrum, future smartphones are expected to support high frequencies like mmWave (the 24–100 GHz range) [38]. Compact antennas arranged as arrays on the PCB can form linear phased arrays with high gain and directional radiation beams [39]. A new, compact phased array mmWave antenna with broad bandwidth and end-fire radiation is introduced, and its fundamental characteristics are investigated. It consists of eight loop-dipole antennas arranged linearly, enabling easy integration into smartphone systems. The properties and performance of the element, as well as the entire MIMO smartphone design and the mmWave array, are meticulously evaluated using CST software 2022 [40]. Through rigorous investigations, the capabilities and efficacy of the proposed antenna system are thoroughly examined, establishing a foundation for an advanced and efficient wireless communication solution for future smartphones.

## 2. Characteristics of the Dual-Polarized CPW-Fed Antenna

**Figure 1:**
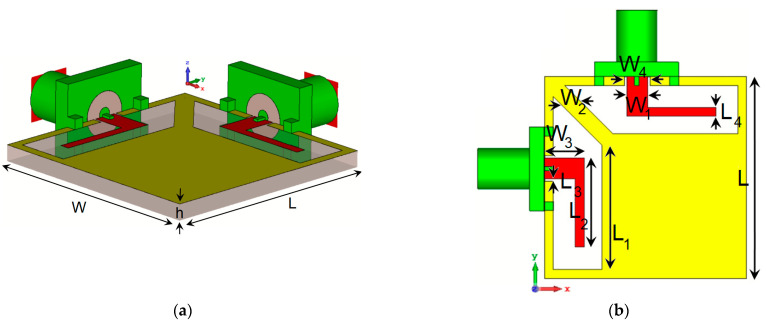
(**a**) Side and (**b**) front views of the introduced antenna design.

This section discusses the critical characteristics of the single-element antenna. The antenna schematic is depicted in Figure 1, featuring two closely positioned trapezoidal slot antennas equipped with L-shaped radiating stubs. The antenna is designed on a substrate made of 1.6 mm FR4 material. This substrate has a permittivity (also known as relative permittivity or ε_r_) of 4.3 and a loss tangent (tan δ) of 0.023. These material properties are crucial in determining the electrical properties and overall performance of the antenna. The choice of the dielectric material influences the antenna’s impedance, radiation efficiency, and bandwidth, among other factors. FR4 is a popular low-cost material commonly used in designing RF and microwave circuits and antennas. The specific dimensions and parameter values of the single and the MIMO antenna designs are outlined in Table 1.

**Figure 2:**
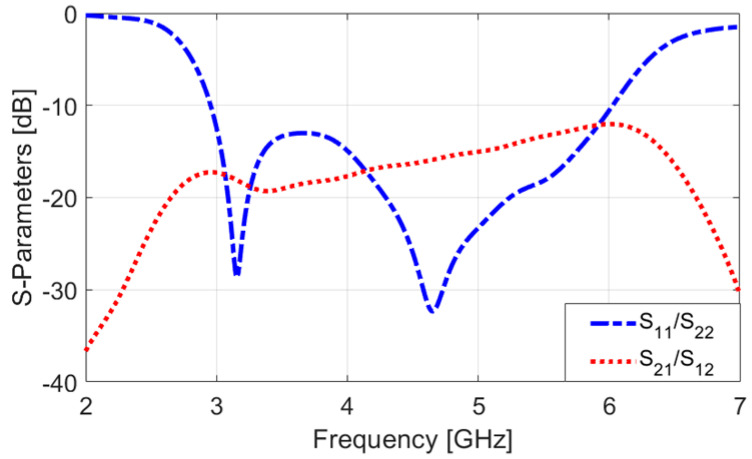
S-parameters of the suggested antenna design.

**Figure 3:**
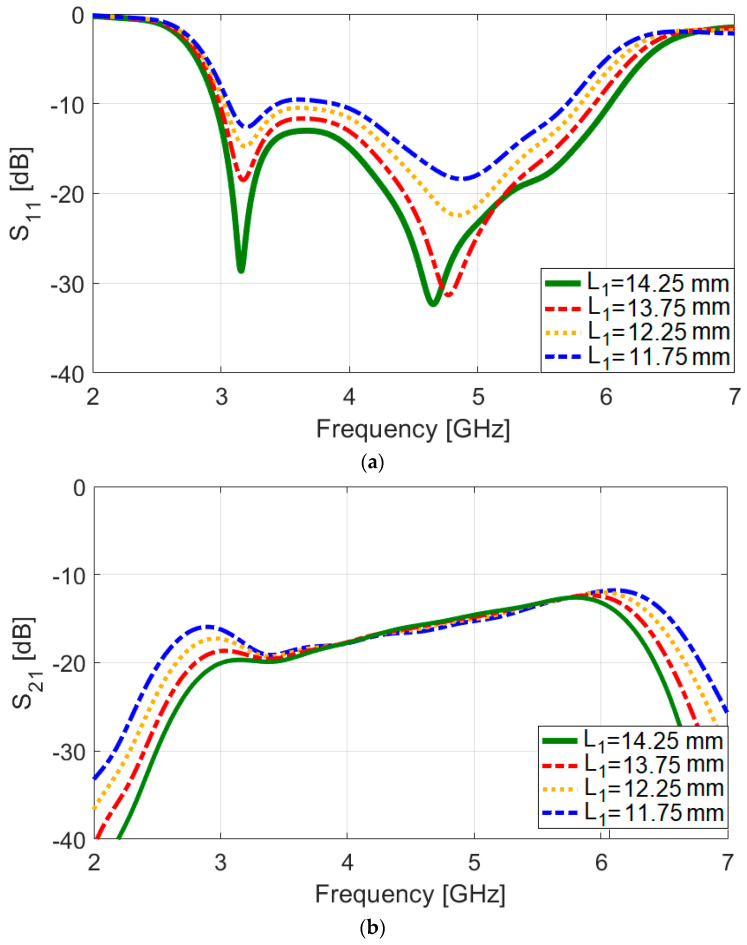
(**a**) S_nn_ and (**b**) S_mn_ results for different values of L_1_.

The S-parameters of the antenna are shown in Figure 2. Notably, the dual-polarized single antenna pair exhibits a wide operational bandwidth from 3.2 GHz to 6 GHz, with an S_nn_ −10 dB. Moreover, the designed CPW-fed antenna demonstrates high isolation, particularly at the center frequency. Figure 3 showcases the antenna’s S-parameters (S_11_/S_21_) with varying L_1_, a specific parameter in the antenna design. During the simulation, L_1_ is adjusted while all other parameters remain constant, as listed in Table 1. The plot demonstrates that different values of L_1_ have a substantial effect on the matching and mutual coupling characteristics between the feeding ports. Notably, for L_1_ = 14.25 mm, the antenna achieves its optimal performance, indicating the best impedance matching and mutual coupling properties among the investigated values of L_1_. As illustrated in Figure 3a, adjusting the value of L_1_ has improved the antenna’s impedance bandwidth, particularly at higher frequencies. Additionally, as shown in Figure 3a, when L_1_ is set to 11.75, the mutual coupling between the elements at the lower frequency (3 GHz) and higher frequency (6 GHz) is approximately −16 dB and −11 dB, respectively. Increasing L_1_ to 14.25 further improves the mutual coupling, reducing it to about −20 dB at 3 GHz and −13 dB at 6 GHz. These demonstrate that adjustments to the L_1_ parameter can influence and reduce mutual coupling within the desired operating band.

**Figure 4:**
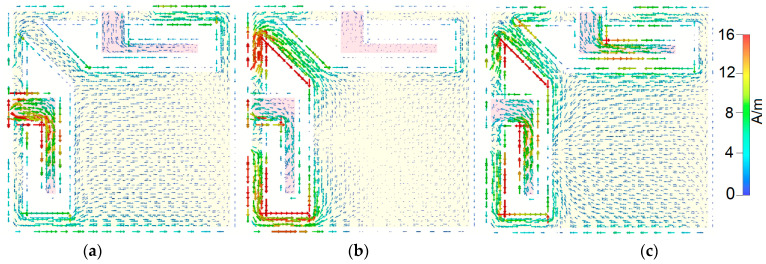
Current densities at (**a**) 3.2, (**b**) 4.7, and (**c**) 5.5 GHz.

**Figure 5:**
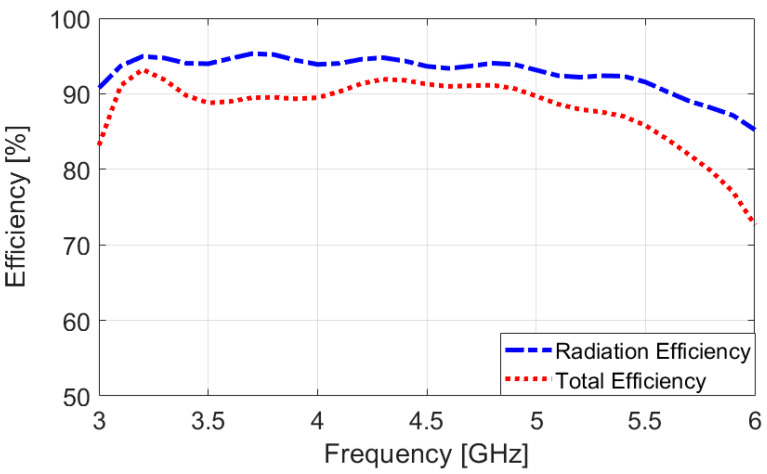
Efficiency results across the antenna’s broad bandwidth.

To better understand the broadband and multi-resonant behavior of the proposed antenna design, Figure 4 presents the surface current densities at 3.2 GHz, 4.7 GHz, and 5.5 GHz. The same maximum scale is applied across all plots for consistency. A detailed examination reveals that significant resonance activity occurs in several key areas as follows: the L-shaped radiation stub, as well as both the inner and outer regions of the trapezoidal slot antenna. These combined contributions from multiple components of the antenna are crucial in achieving the wide bandwidth performance observed. This synergy between the design elements highlights the antenna’s effectiveness in generating broad-spectrum resonances [41,42]. Figure 5 presents the efficiency characteristics of the dual-polarized antenna, including both the radiation efficiency (R.E.) and total efficiency (T.E.). The antenna demonstrates impressive efficiency, achieving over 80% efficiencies across a frequency range from 3 to 6 GHz. This high efficiency indicates that a substantial fraction of the input power is emitted as electromagnetic radiation, making the antenna an effective and capable performer within the specified frequency band. Such efficiency levels reflect the antenna’s strong potential for practical applications in modern systems.

**Figure 6:**
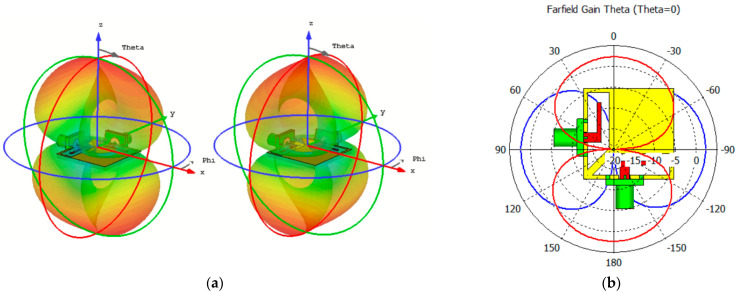
Linear-scaled (**a**) 3D and (**b**) 2D dual-polarized radiation patterns at 4.5 GHz.

**Figure 7:**
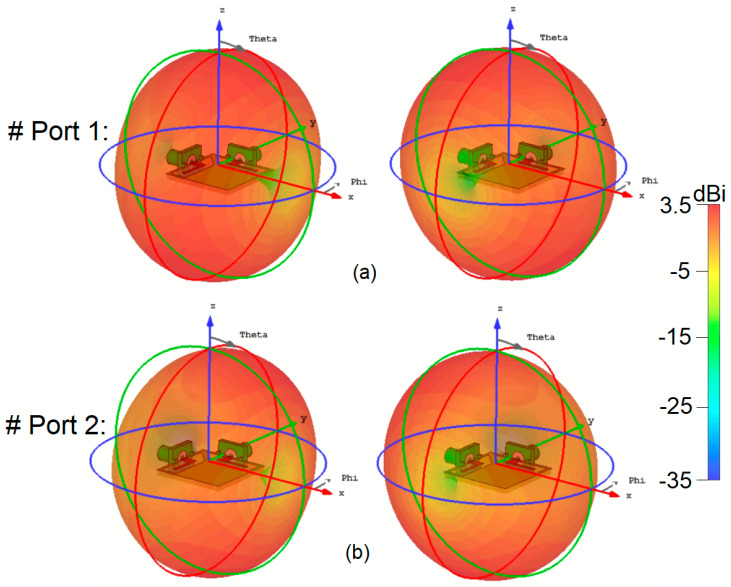
3D radiation patterns of the dual-polarized antenna at (**a**) 3.5 and (**b**) 5.5 GHz.

In Figure 6a, the linear-scaled radiation patterns (Phi) for the various antenna ports are depicted. The figure reveals that the dual-polarized antenna exhibits identical radiation patterns from these ports. In Figure 6b, similar 2D radiation performances are clearly observed, with a 90° phase difference and polarization diversity resulting from the different arrangements of the ports. The antenna’s design allows it to maintain consistent radiation characteristics across its dual-polarized nature, providing reliable and balanced performance regardless of the feeding port arrangement. Figure 7 demonstrates the 3D radiation patterns of the antenna at 3.5 and 5.5 GHz. It is evident that the antenna exhibits similar radiation patterns with distinct orthogonal polarizations. Additionally, the antenna gain, which refers to the actual power gain realized by the antenna in a specific direction, exceeds 3 dB. This indicates that the antenna achieves good directional performance and efficiently radiates electromagnetic waves when fed from both Port 1 and Port 2, offering a balanced and effective radiation pattern for diverse applications [43,44]. It is worth noting that the antenna’s 3–4 dBi gain meets the typical smartphone requirements, prioritizing omnidirectional coverage over high gain. For sub-6 GHz applications requiring higher gain, directional antennas such as patch or end-fire designs (e.g., Yagi or Vivaldi) are potential options. However, their larger size and limited coverage make them less suitable for compact smartphone designs. Array antennas can also enhance gain but are more commonly employed in higher frequency mmWave smartphone systems, where smaller element sizes are practical. This is discussed further in Section 6.

## 3. Fundamental Characteristics of the Suggested MIMO Antenna

**Figure 8:**
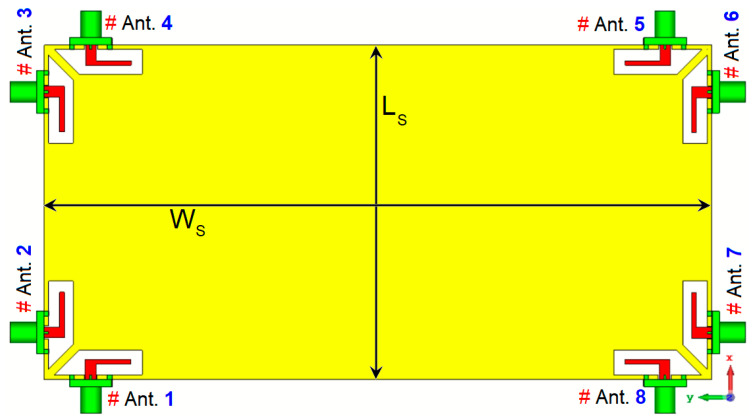
Schematic of the introduced MIMO antenna.

**Figure 9:**
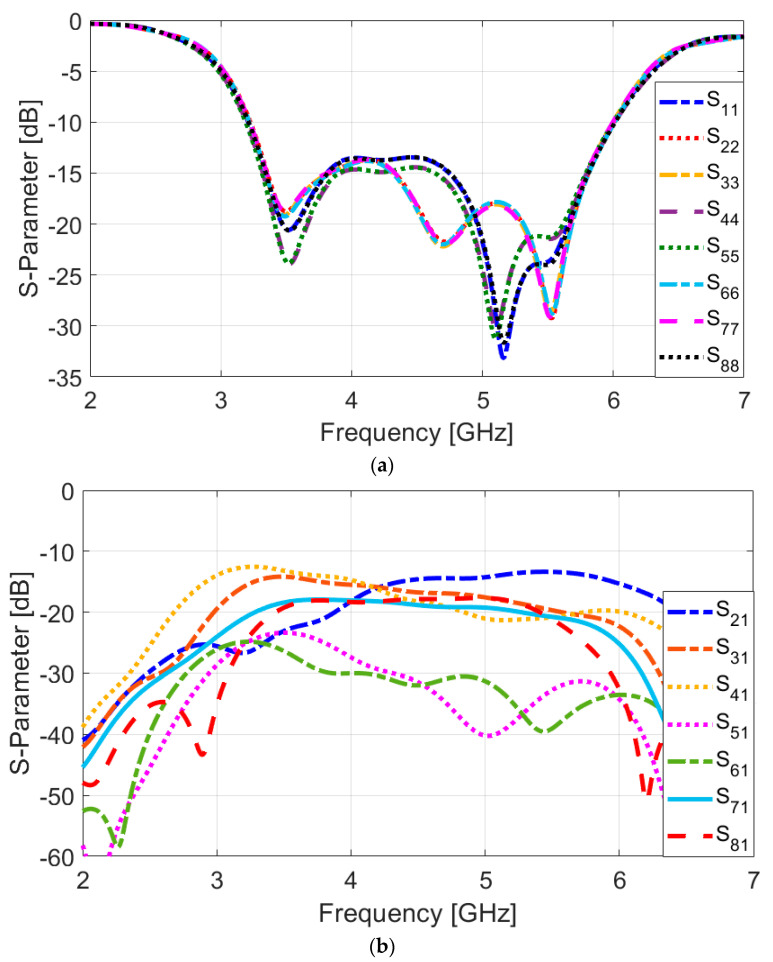
(**a**) S_nn_ and (**b**) S_mn_ results of the antenna elements.

Figure 8 depicts the schematic of the investigated MIMO antenna design. The structural layout of this multi-feed antenna system is relatively simple and straightforward. The antenna’s overall dimension is 75 × 150 mm^2^, and it includes an array of 8 × 8 trapezoidal slot antennas, each paired with L-shaped stubs and CPW feeds, all positioned in close proximity to one another. It is worth mentioning that multiple similar antenna elements are positioned at PCB’s edges. The utilization of the suggested antennas has several advantages. Firstly, it enhances the frequency response and matching characteristics of the antenna. Secondly, it results in symmetrical radiation patterns that cover both/bottom areas of the board. This symmetrical radiation pattern is desirable as it provides better overall coverage and performance for the MIMO system. Overall, the design aims to achieve efficient and reliable performance for smartphone applications, considering the compact size and appropriate radiation characteristics [45,46]. Figure 9 presents the S-parameters for the designed antenna array, demonstrating that the antenna elements exhibit consistent performance across the frequency range. The reflection coefficients (S_nn_) are notably low, with values better than −15 dB within the broad operational frequency band from 3.2 to 6 GHz, indicating excellent matching. Additionally, as depicted in Figure 9b, the mutual coupling between the elements is effectively minimized, with isolation levels (S_nm_) exceeding −13 dB. This indicates that the antenna elements maintain strong isolation from each other, contributing to improved overall performance and reduced interference. Typically, values as low as −10 dB, and in some cases even −6 dB, are considered standard and acceptable isolation levels in smartphone MIMO antenna designs. However, it is worth mentioning that to further enhance isolation between the antenna elements, various decoupling techniques such as applying additional parasitic elements, using metamaterials, introducing neutralization networks, or implementing electromagnetic bandgap (EBG) can be employed [47]. They might, however, increase the complexity and size of the antenna, which is important considering the limited space occupied by antennas in smartphone boards.

**Figure 10:**
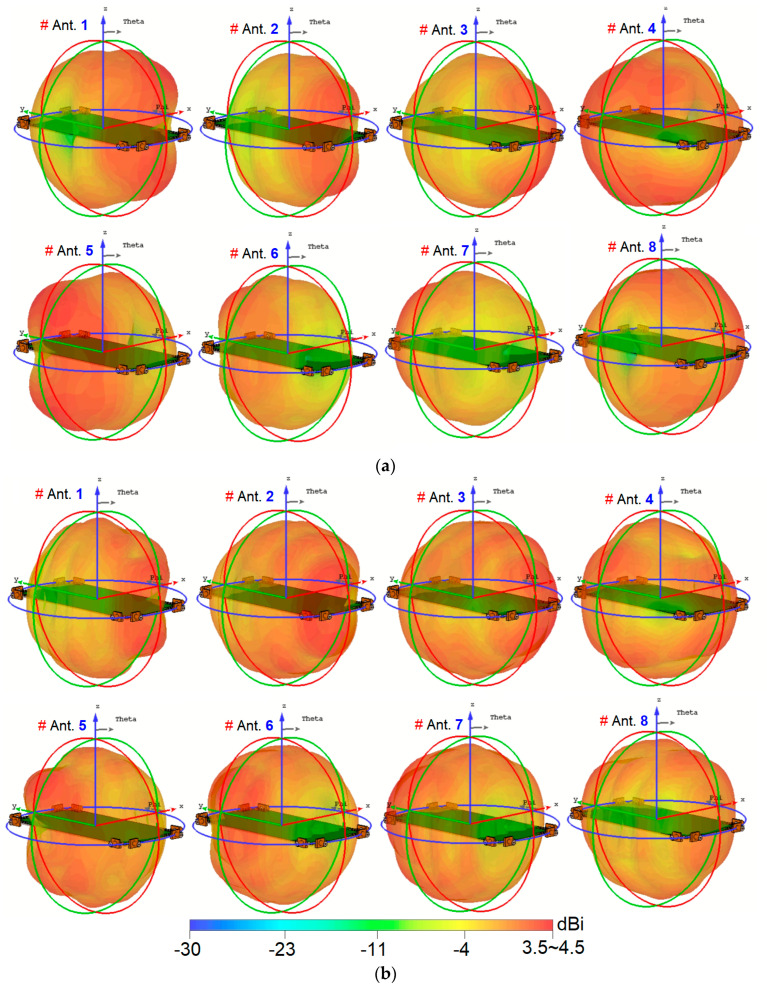
Three-dimensional radiation patterns at (**a**) 3.5 GHz and (**b**) 5.5 GHz.

In Figure 10, the 3D radiation patterns of the multiple elements, along with their corresponding gain values at different frequencies, including 3.5 and 5.5 GHz, are displayed. The figure reveals that the 8-antenna system provides radiation with various polarizations. It is important to note that the observation of polarization diversity stems from the placement and orientation of the antenna elements, as clarified in Figure 6a. In the smartphone MIMO antenna layout, adjacent elements are positioned at 90-degree angles to each other. This polarization diversity applies to all antenna pairs in the array, enabling the system to support multiple polarization states, which is reflected in the overall radiation performance illustrated in Figure 10. Moreover, the gain levels are sufficient to cover the different regions of the smartphone board effectively. This observation suggests that the investigated array design is robust concerning the holding positions of 5G handheld devices. Regardless of how users hold their smartphones, the antenna array maintains reliable and strong signal reception and transmission due to its multi-antenna configuration and diverse radiation patterns. This characteristic is essential in 5G smartphones to ensure consistent and stable connectivity in various usage scenarios and positions [48,49,50].

**Figure 11:**
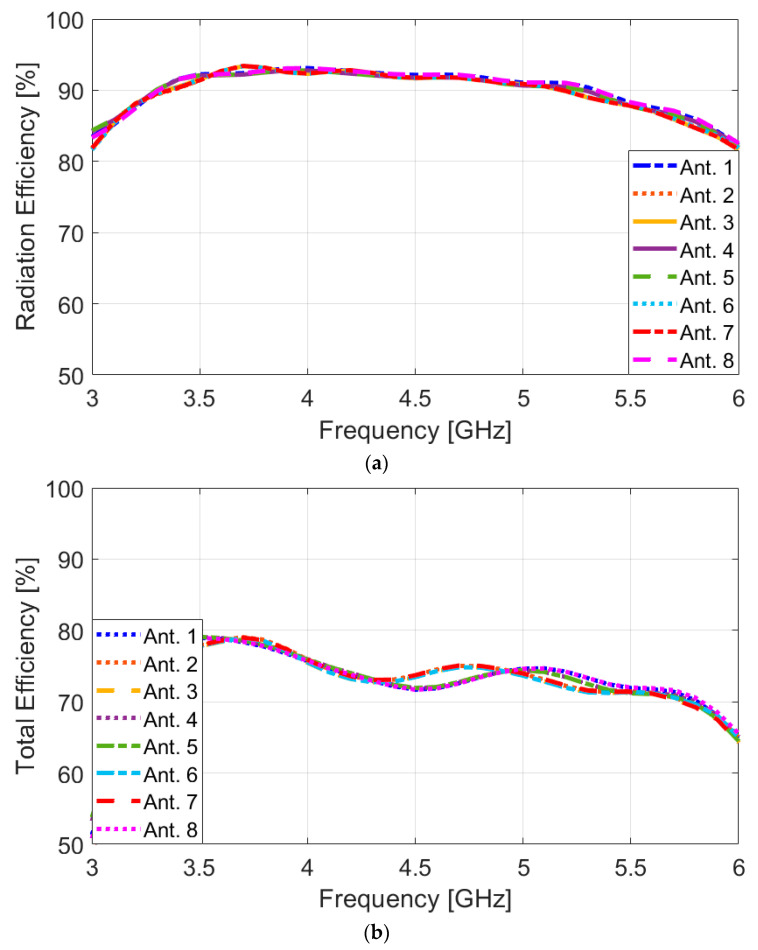
(**a**) Radiation and (**b**) total efficiency results of the elements over 3–6 GHz.

In Figure 11, the efficiency characteristics of the MIMO design are represented. Figure 11a illustrates the high radiation efficiency of each antenna element, surpassing 80%. Additionally, it can be observed that these elements exhibit total efficiencies exceeding 70% over most of the operational frequency range. The achieved efficiencies are deemed quite satisfactory for smartphone operation, indicating that the introduced MIMO antenna design performs efficiently in practical scenarios. High radiation efficiency ensures that a significant portion of the input power is converted into electromagnetic waves, leading to effective signal transmission and reception. Moreover, the total efficiency considers all losses and mismatches in the system, reflecting the effectiveness of the antenna in real-world usage [51,52].

## 4. Prototyping and Measurements of the Suggested Design

**Figure 12:**
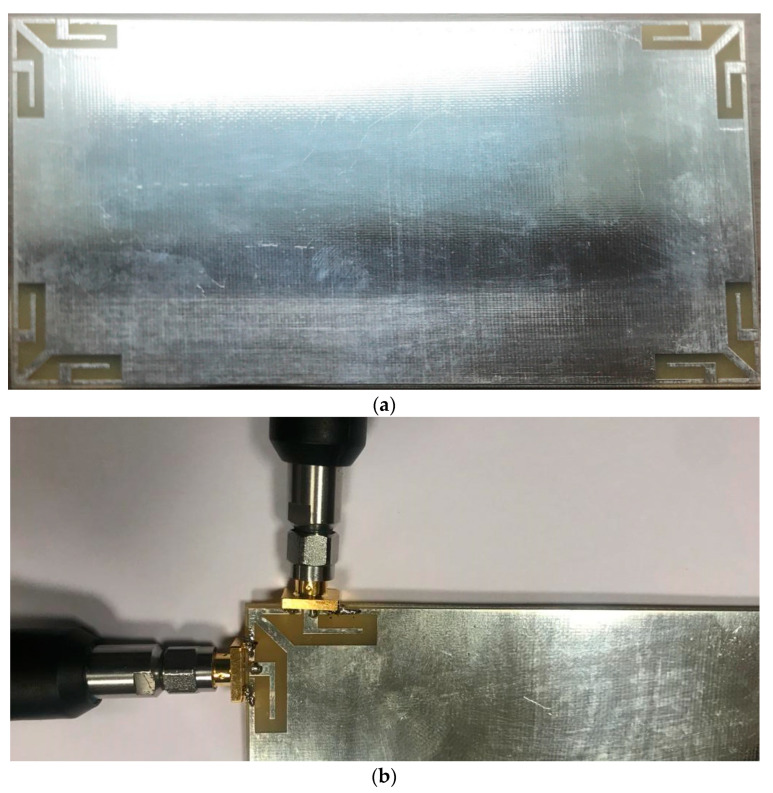
(**a**) Prototyped sample and (**b**) feeding method.

In this section, the fabricated prototype sample of the MIMO antenna design is presented, along with measurement results and comparisons. Figure 12a displays a photograph of the prototype sample. The antenna is implemented on a single side of a low-cost FR4 substrate and features an 8 × 8 MIMO configuration. Figure 12b illustrates the feeding method used for the antenna pair. The SMA connectors’ inner conductors are soldered to the CPW antennas, while the outer connectors are attached to the ground plane. This configuration ensures effective signal transmission and reception between the antenna elements and the measurement equipment, optimizing performance and reliability in the testing process.

**Figure 13:**
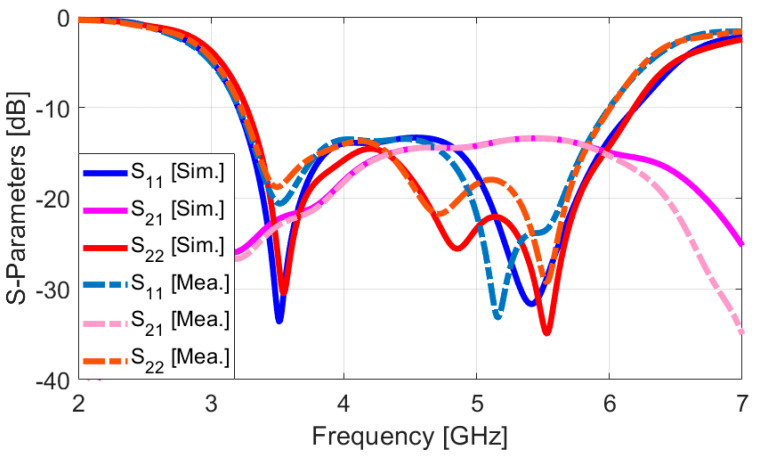
Measured/simulated S-parameters.

**Figure 14:**
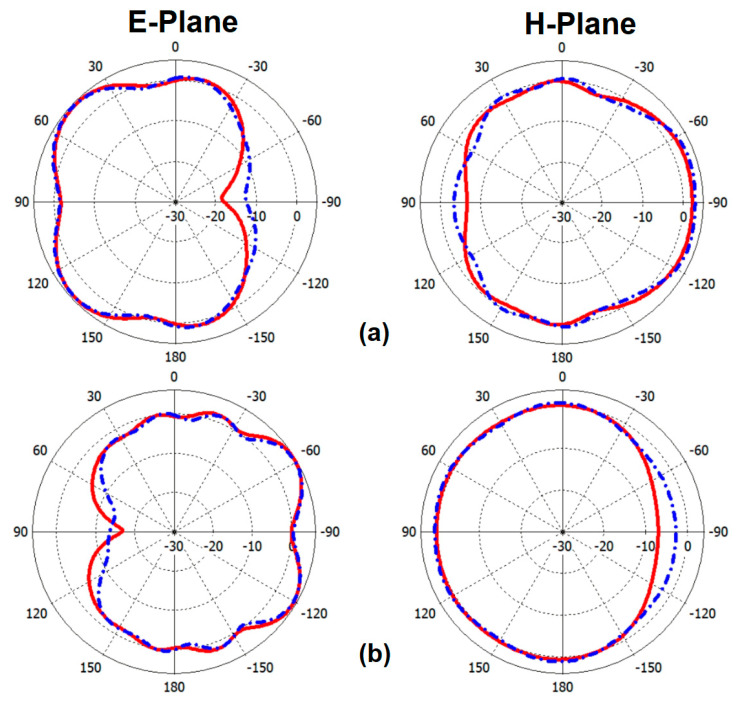
Measured/simulated 2D radiation patterns of the pair at (**a**) 3.5 GHz and (**b**) 5.5 GHz.

In the following analysis, the performance characteristics of the smartphone array design for Ports 1 and 2 are assessed and compared, due to their similar placements and the overall characteristics of the antenna pairs. Figure 13 displays the measured and simulated S-parameter results. It is evident from the figure that the measured and simulated S_11_/S_22_ values align well, particularly in covering the necessary multi-operation bands. The antenna design demonstrates an impressive impedance bandwidth, with S11 values below −10 dB, spanning from 3.2 GHz to over 6 GHz. This broad bandwidth indicates that the antenna is well-suited for a variety of frequency applications. Moreover, the mutual couplings (S_21_) between the adjacent antenna elements are measured to be less than −14 dB at the desired frequency band. This indicates that the interference between the elements is effectively minimized, ensuring the proper isolation and individual performance of each antenna in the MIMO array [53]. Figure 14 presents the 2D radiation patterns (H-plane and E-plane) of the antenna elements, both from measurements and simulations, at frequencies of 3.5 GHz and 5.5 GHz. During the measurement process, one port of the antenna was excited while the other was terminated with a 50 Ω load to ensure precise characterization. The resulting radiation patterns reveal that the prototype of the sample handset antenna demonstrates effective quasi-omnidirectional radiation performance across these resonance frequencies, indicating its suitability for applications requiring broad coverage. This means that the radiation of the antenna has been distributed relatively evenly in various directions around the element, making it ideal for practical mobile communication scenarios where users may hold their devices in different orientations [54,55,56].

To evaluate the MIMO capability of the presented array, two important parameters, the ECC and the TARC properties, are examined to ensure that the antenna array provides optimal performance in terms of signal diversity and efficiency, which are critical for effective MIMO operation [57]. These parameters are determined using the following formula:

(1) $$ECC=\frac{{\left|S_{mm}^{*}S_{mn}+S_{nm}^{*}S_{nn}\right|}^{2}}{{\left(1-{\left|S_{mm}\right|}^{2}-{\left|S_{mn}\right|}^{2}\right)\left(1-{\left|S_{nm}\right|}^{2}-{\left|S_{nn}\right|}^{2}\right)}^{*}}$$

(2) $$TARC=-\sqrt{\frac{{\left(S_{mm}+S_{mn}\right)}^{2}+{\left(S_{nm}+S_{nn}\right)}^{2}}{2}}$$

**Figure 15:**
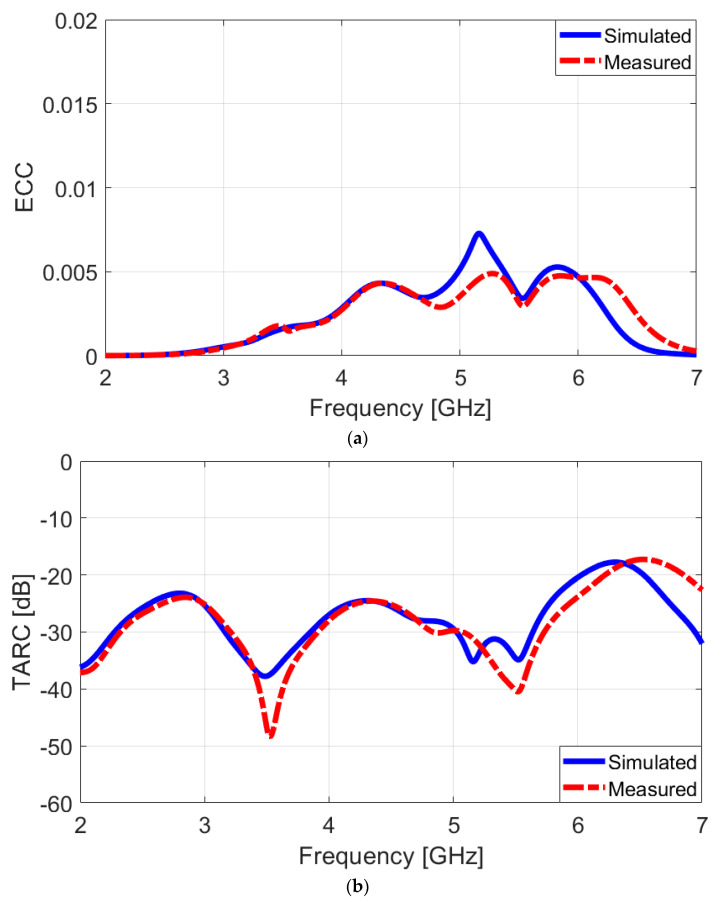
Measured/simulated comparison for (**a**) ECC and (**b**) TARC.

Figure 15a,b depicts the calculated ECC/TARC characteristics, respectively. The results show that the ECC is quite low, measuring less than 0.005, and the TARC is below −20 dB, both taken from within the target frequency band. A low ECC value indicates that the signals received or transmitted by the different elements exhibit minimal correlation. This is desirable in MIMO systems as it reduces interference and enhances the antenna array’s ability to provide independent and diverse communication paths. Similarly, a low TARC value implies that the antenna array efficiently radiates the input power, resulting in little to no reflection back to the input ports. The presented ECC and TARC demonstrate that the antenna array is highly compatible with MIMO functionality, as it maintains low correlation and optimal radiation efficiency across the target band, providing reliable and effective performance for multi-antenna scenarios.

## 5. SAR Evaluation

**Figure 16:**
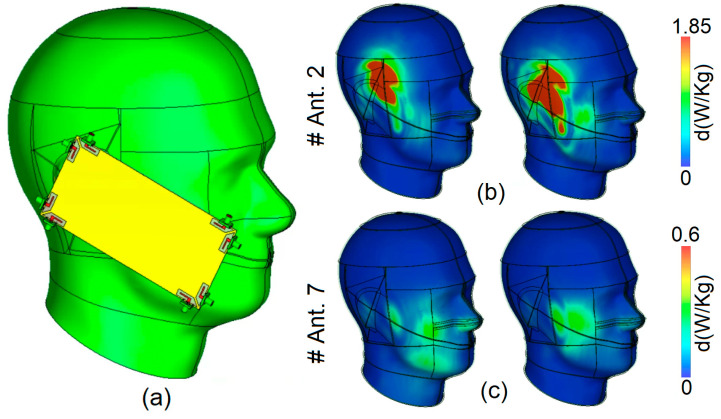
(**a**) Talk-mode and SAR analysis at (**b**) 3.5 GHz and (**c**) 5.5 GHz.

The Specific Absorption Rate (SAR) measures the energy absorbed by the human body upon exposure to radio frequency (RF) electromagnetic fields. It is presented in watts per kilogram (W/kg), and it indicates the rate at which energy is absorbed by tissue. It is a critical metric for ensuring that electronic devices, particularly mobile phones, comply with safety standards to minimize potential health risks [58]. SAR values are influenced by device design, usage scenarios, and environmental conditions. Regulatory bodies like the FCC and ICNIRP have established SAR limits to protect public health, typically set at 2 W/kg, to prevent excessive energy absorption that can lead to tissue heating [59]. The SAR distribution for the selected antenna elements in talk-mode scenarios has been analyzed and is illustrated in Figure 16. This figure highlights the antenna elements with both the highest and lowest SAR levels observed in the scenarios. Specifically, Figure 16 reveals that, during talk mode, Antenna 2 exhibits the highest SAR level, whereas Antenna 7 shows the lowest SAR value. This variation is linked to the arrangement of the antenna array as follows: Antenna 2 is positioned closer to the hand phantom compared to Antenna 7. Consequently, it can be inferred that a shorter distance between the antennas and the phantom results in an elevated SAR level, while a greater distance corresponds to a lower SAR level.

## 6. Comparison

Table 2 provides a performance comparison among the newly introduced MIMO array and various other designs discussed in the existing literature. Various fundamental properties, such as the employed antennas, efficiency, Envelope Correlation Coefficient (ECC), among others, are discussed for each design. The results of the comparison reveal that the developed array exhibits improved functionalities and offers several advantageous characteristics, particularly in terms of the operational frequency band. The designed array offers an impressive frequency bandwidth of more than 2800 MHz, making it capable of covering a wide range of sub-6 GHz frequencies. This broad bandwidth is crucial for supporting various 5G applications that require high data rates and reliable connectivity. An additional advantage of the presented antenna array is its planar schematic and ease of integration on a single-layer substrate. Furthermore, the presented antenna provides a diversity function in terms of both pattern and polarization characteristics, offering full coverage while supporting various sides of the board. These significant advantages position the proposed antenna as a competitive and practical solution for enabling advanced 5G communication technologies.

## 7. Possible Integration of a High-Frequency Antenna

**Figure 17:**
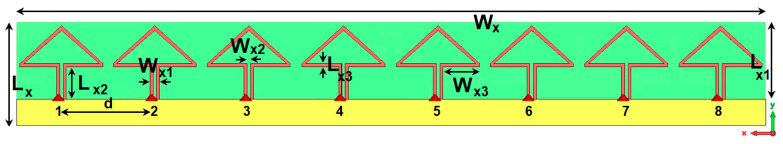
Schematic of the phased array.

**Figure 18:**
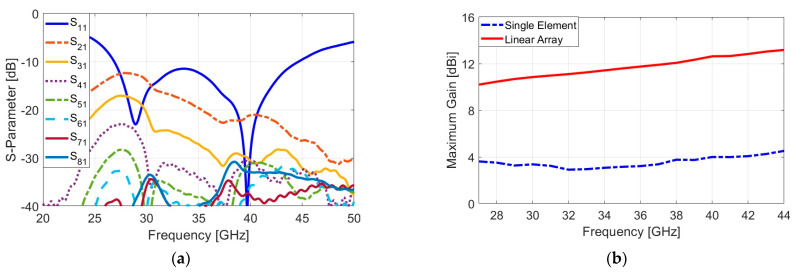
(**a**) S-parameters and (**b**) gain comparison of the designed phased array.

Beyond the sub-6 GHz spectrum, integrating high-frequency antennas, such as mmWave phased arrays, is crucial for advancing 5G and paving the way for future 6G networks [60]. As proof of concept, a new mmWave phased array antenna has been developed for integration into shared smartphone PCBs. The configuration and design details are shown in Figure 17, featuring eight end-fire loop-dipole antenna elements that are linearly arranged on a compact board with overall dimension W_X_ = 36, L_X_. The parameter values in millimeters are as follows: W_X_ = 36, L_X_ = 5, W_X1_ = 0.4, L_X1_ = 3.25, W_X2_ = 0.15, L_X2_ = 1.35, W_X3_ = 1.5, and L_X3_ = 0.15. Figure 18a shows the S-parameters of the array, demonstrating a broad impedance bandwidth of 27–44 GHz, supporting key mmWave frequencies (such as 28, 36, 38, and 45 GHz) with adequate mutual coupling (<−13 dB) between elements. Figure 18b compares the maximum gains of a single antenna element and the phased array over the operating frequency band. The individual element achieves gains of 4–5 dBi, while the phased array significantly outperforms this, with gains exceeding 12 dBi.

**Figure 19:**
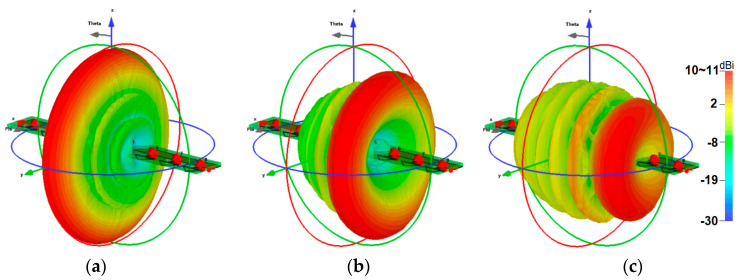
Beam-steering of the phased array at (**a**) 0, (**b**) 30, and (**c**) 60 degrees.

**Figure 20:**
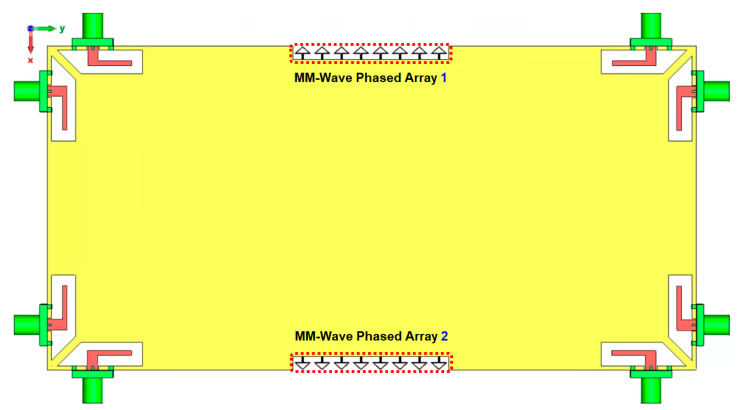
Possible placements of the mmWave phased array.

Figure 19 further illustrates the 3D beam-steering functionality of the phased array at 30 GHz, showing robust beam-steering capabilities with end-fire radiation patterns and high gain levels. These results underscore the array’s potential for enhanced directional communication and connectivity in mmWave 5G and future 6G applications. Figure 20 illustrates the potential placements of the proposed mmWave phased array within the smartphone PCB configuration. Its compact design allows for seamless integration into small areas of the board, demonstrating versatility and adaptability. Further investigation could be considered in future research to fully unlock the potential of high frequency antennas for 5G and 6G technologies.

## 8. Conclusions

This paper introduces a new dual-polarized eight-antenna array design that employs CPW-fed trapezoid slot antennas with L-shaped feedings. The primary goal of this design was to achieve a wide/broad bandwidth from 3.2 to 6 GHz to accommodate the 5G spectra. The presented MIMO design is implemented on a single layer of a smartphone board, utilizing the cost-effective and widely used FR4 substrate material, without compromising its overall performance. The results demonstrate that the proposed smartphone antenna design outperforms recently reported designs in several key aspects. It exhibits superior efficiency results, improved coverage, minimal ECC/TARC parameters, and a notably wider impedance bandwidth. Furthermore, due to its single-layer planar structure without ohmic losses, the design proves to be highly promising for future handheld platforms. The experimental results of the suggested design align well with the simulation data, affirming its reliability and validity. These compelling features make the proposed design an ideal candidate for high data-rate mobile networks in future smartphones. Overall, this dual-polarized/eight-antenna model presents a significant advancement in MIMO smartphone antenna technology, offering enhanced performance and broader applications in the rapidly evolving world of mobile communications. Moreover, a compact mmWave phased array antenna with broad bandwidth is presented, and its properties are discussed, highlighting its potential for future smartphone connectivity.

## Figures and Tables

**Table 1:** Design values of the antenna parameters.

Param.	mm	Param.	mm
W	23	W_2_	1.8
L	23	L_2_	10.2
W_S_	150	W_3_	4.5
L_S_	75	L_3_	0.25
W_1_	2.4	W_4_	2.9
L_1_	14.25	L_4_	1

**Table 2:** Comparison table of the suggested broadband MIMO antenna.

Ref.	Antenna Trype	Bandwidth (GHz)	Efficiency (%)	Size (mm^2^)	Isolation (dB)	ECC	Diversity
[10]	Monopole-slot	2.55–2.65 (0.1)	50–70	136 × 68	12	<0.15	Yes
[11]	L-shaped monopole	3.4–3.6 (0.2)	50–5	136 × 68	15	<0.10	No
[12]	Self-isolated monopole	3.4–3.6 (0.2)	60–70	150 × 75	19	<0.02	No
[13]	Balanced open-slot	3.4–3.6 (0.2)	60–75	150 × 80	17	<0.05	No
[14]	Inverted-F antenna	3.4–3.6 (0.2)	-	110 × 60	19	-	Yes (Limited)
[15]	Decoupled open-slot	3.4–3.6 (0.2)	40–60	150 × 75	12	<0.40	No
[16]	Rectangular Slot	3.4–3.6 (0.2)	50–60	136 × 68	11	c < 0.05	No
[17]	H-shaped monopole	3.4–3.6 (0.2)	60–70	150 × 75	11	<0.15	No
[18]	Gap-Coupled Loop	3.4–3.6 (0.2)	40–60	150 × 75	12	<0.20	No
[19]	S-shaped monopole	3.45–3.55 (0.1)	50–68	130 × 50	15	<0.30	No
[20]	Shorted loop	3.4–3.6 (0.2)	50–78	150 × 77	10	<0.1	No
[21]	L-shaped monopole	3.4–3.6 (0.2)	40–70	145 × 75	15	<0.15	No
[22]	L-shaped strip monopole	3.4–3.6 (0.2)	40–70	150 × 63	10	<0.1	No
[23]	Folded dipole	3.45–3.55 (0.1)	50–70	154 × 74	15	<0.1	No
[24]	Inverted-F antenna	3.3–3.8 (0.5)	40–75	150 × 75	13	<0.06	No
[25]	Corner-cut patch	4.4–5 (0.6)	40–80	150 × 75	10	<0.1	No
[26]	Square-loop with slot	3.4–3.8 (0.4)	55–70	150 × 75	17	<0.03	Yes
[27]	E-shaped slot	3.4–3.6 (0.2)	40–75	150 × 75	19	<0.1	No
[28]	L-shaped slot	3.6–4.7 (1.1)	85–95	150 × 75	10	<0.08	No
[29]	SCS Patch-Slot	3.5–3.7 (0.2)	50–80	150 × 75	16	<0.03	Yes
[30]	Coupled-loop monopole	3.3–5 (1.7)	40–70	150 × 75	13	<0.1	No
[31]	E-shaped slot with F-probe	3.5/4.7 (0.2/0.2)	65–70	150 × 80	18	<0.1	Yes (Limited)
[32]	PIFA	3.8/5.4 (0.1/0.1)	40–85	150 × 74	13	<0.01	No
[33]	Monopole with T-slot	3.5/5.5(0.4/1)	30–80	150 × 75	14	<0.02	No
[34]	Open-loop monopole	3.5/4.9 (0.2/0.2)	60–80	150 × 75	10	<0.1	No
[35]	Paired Slot	3.4–3.6 (0.2)	40–65	150 × 75	18	<0.1	No
**This Work**	**Trapezoid slot with L-monopole feeding**	**3.2–6 (2.8)**	**65–80**	**150 × 75**	**13**	**<0.005**	**Yes**

## Data Availability

The original contributions presented in this study are included in the article. Further inquiries can be directed to the corresponding authors.
